# The Dilemma of Delayed Replantation of an Avulsed Tooth—Case Presentation

**DOI:** 10.1155/crid/2244600

**Published:** 2025-11-03

**Authors:** Mihaela Chirila, Ioana Suciu, Ecaterina Ionescu, Lucian Chirila, Dana Bodnar, Adriana Vasilache

**Affiliations:** ^1^Department of Endodontics, Faculty of Dentistry, University of Medicine and Pharmacy “Carol Davila”, Bucharest, Romania; ^2^Department of Orthodontics and Dentofacial Orthopedics, Faculty of Dentistry, University of Medicine and Pharmacy “Carol Davila”, Bucharest, Romania; ^3^Department of Oro-Maxillo-Facial Surgery, Faculty of Dentistry, University of Medicine and Pharmacy “Carol Davila”, Bucharest, Romania; ^4^Department of Restorative Odontotherapy, Faculty of Dentistry, University of Medicine and Pharmacy “Carol Davila”, Bucharest, Romania

**Keywords:** delayed replanting, dental avulsion, informed consent

## Abstract

Dental avulsion is one of the most severe forms of dental trauma, often requiring urgent intervention. The prognosis of replantation depends heavily on the extraoral time and the storage conditions of the avulsed tooth. This study is aimed at presenting and analyzing a clinical case of delayed replantation of a maxillary central incisor in a prepubertal patient, highlighting the therapeutic decisions, challenges, and long-term outcomes. A 14-year-old patient suffered avulsion of two maxillary incisors during a sports accident. One tooth was recovered 22 h posttrauma and stored in milk before replantation. Endodontic treatment was performed ex vivo, and the tooth was replanted 30 h after the incident. Despite initial clinical success, radiographic signs of inflammatory root resorption were observed at the 1-year follow-up. This case demonstrates that although delayed replantation carries a high risk of complications such as root resorption and ankylosis, it remains a viable option, especially in young patients due to its role in preserving alveolar bone and mitigating psychological and esthetic impacts.

## 1. Introduction

Dental injuries are unintended impacts that can affect the teeth and surrounding structures, including the periodontium, alveolar process, maxillary bones, and soft tissues around the mouth. Dental avulsion is a serious type of traumatic injury that involves the tearing of the pulpal neurovascular bundle and the periodontal ligament (PDL), causing the tooth to separate from the dental alveolus and to be exposed to the outside environment [1]. Although the ideal course of action is to immediately replant the avulsed tooth, this may not always be feasible. The conditions present at the accident site, along with insufficient understanding of how to manage avulsion, are significant factors in this situation. Dental injuries can lead to various effects on the affected individual, their family, and the wider community [2]. Some age demographics are more susceptible, yet anyone can face the risk of trauma through routine activities [3]. The effects of dental trauma extend beyond just physical damage, impacting psychological and social aspects as well.

Reimplantation of teeth that have been completely knocked out is a critical dental procedure that falls under maximum emergency care. This procedure is performed following the management of the patient's neurological and overall emergency needs. If the patient is stable and awake, obtaining informed consent is essential and obligatory. Based on the patient's age, consent from a parent or legal guardian is necessary. According to the patient's age, it is necessary to obtain informed consent from the parents or legal guardians. The optimal outcome for an avulsed tooth is largely influenced by how quickly treatment is administered, the duration the tooth remains outside of the alveolar socket, and the conditions (whether physiological or nonphysiological) in which the avulsed tooth is preserved. In this context, awareness campaigns regarding the correct immediate response in the event of trauma are crucial.

The objective of this work is to present and analyze a clinical case of delayed replantation of a maxillary central incisor in a prepubertal patient, highlighting the therapeutic decisions, challenges, and long-term outcomes. The study was approved by the research ethics committee (Protocol Number 24585/11.09.2023) of the University of Medicine and Pharmacy “Carol Davila”, Bucharest.

## 2. Case Presentation

A 14-year-old patient came to the clinic following an injury from a sports incident. According to the history provided, he experienced a slip accident 18 h prior while playing soccer with friends in the rain. During a crucial moment in the game, he lost his footing on the wet grass and collided head-on with one of the goal posts. Following the jolt of the collision, he realized that he had lost two front teeth, which he searched for in the grass alongside his friends. With poor visibility caused by the rain and fading light, he decided to head home. The following day, he went in for a quick denture fitting.

**Figure 1:**
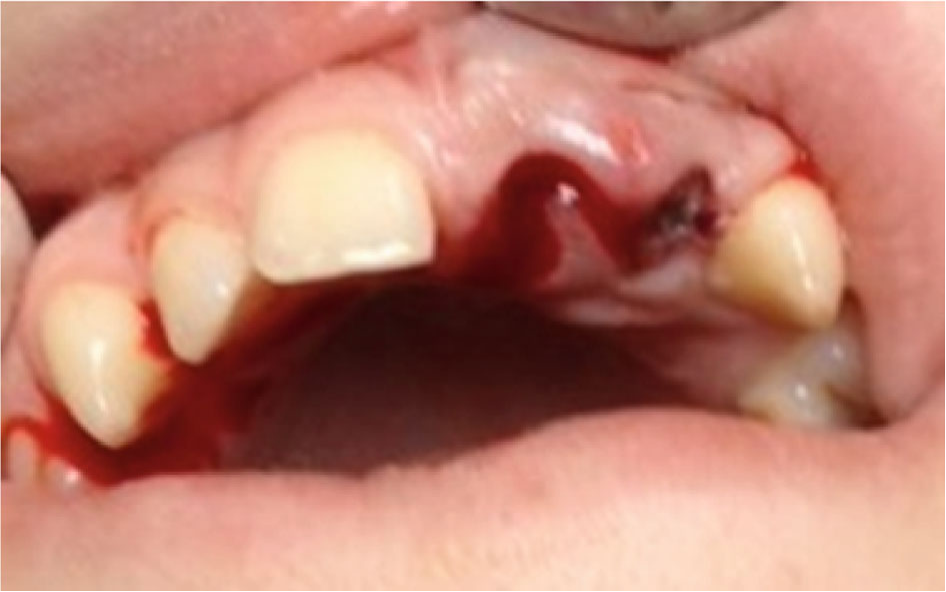
Intraoral aspect at the time of presentation. Alveolar wounds due to the absence of Teeth 1.1 and 1.2.

During the extraoral evaluation, bruising is noted on the upper lip. In the intraoral assessment, the empty sockets of Incisors 2.1 and 2.2 are identified (Figure 1). Both extraoral and intraoral examinations are conducted. There are no intraoral lacerations, fractures of the alveolar walls or bone, nor are there any other teeth affected by the trauma.

At the extraoral examination, ecchymosis was observed in the upper lip region. Intraoral evaluation revealed empty alveoli corresponding to Teeth 2.1 and 2.2 (Figure 1). No additional intraoral lesions, fractures of the alveolar walls or bone, or involvement of other teeth were noted. Radiographic imaging did not yield any further diagnostic information. Based on these findings, a diagnosis of dental avulsion for Teeth 2.1 and 2.2 was established. A tetanus booster was administered, and a neurological examination was advised. Although the patient reported no loss of consciousness, his complaint of facial fatigue suggested possible sleep deprivation from the previous night.

Three hours later, the patient called to report that, accompanied by a friend, he had resumed his search and recovered one of the avulsed teeth—specifically Tooth 2.1, approximately 22 h after the accident. During this interval, continuous heavy rainfall ensured that the tooth remained hydrated; however, the storage medium was nonphysiological and posed an increased risk of bacterial contamination. From the time the tooth was retrieved until its delivery to the clinic, it was preserved in milk. The visible joy and relief of the patient upon recovering even one of the two avulsed teeth was remarkable. Once in the clinic, the tooth was gently cleaned to remove dust and remnants of necrotic PDL tissue and then stored in an amoxicillin/clavulanic acid solution (1000 mg/200 mg) until replantation.

**Figure 2:**
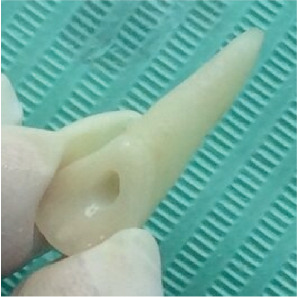
The appearance of Tooth 2.1 after the completion of endodontic treatment before clogging the canal with calcium hydroxide.

Thirty hours after the accident, the recovered tooth (Tooth 2.1) was removed from its storage medium (milk/antibiotic solution), thoroughly irrigated with saline, and inspected for replantation. Clinical and microscopic evaluations performed using an operating microscope confirmed that Tooth 2.1 was intact, exhibited a fully formed root, and presented an eccentrically positioned wide apical orifice with no evidence of cracks or fractures at the coronal or root level. Necrotic PDL remnants were gently removed by wiping with gauze moistened with saline. Due to the prolonged extraoral period, pulpal vitality was deemed compromised, prompting the decision to perform ex vivo endodontic treatment. The dental pulp was carefully extirpated, and a glide path was established using stainless steel files. The tooth was manipulated gently by hand to minimize unnecessary contact with the root surface. Mechanical preparation was executed with rotary instrumentation using ProTaper Universal files (S1–F5) according to the manufacturer's instructions (Figure 2). During instrumentation, the canal was irrigated with 2 mL increments of 1% sodium hypochlorite after each file change. Upon completion of the cleaning and shaping, the root canal was dried and sealed with calcium hydroxide, and the access cavity was restored with glass ionomer cement. The prepared tooth was then maintained in a 2% sodium fluoride solution until the patient was ready for the replantation procedure.

### 2.1. Surgical Stage

**Figure 3:**
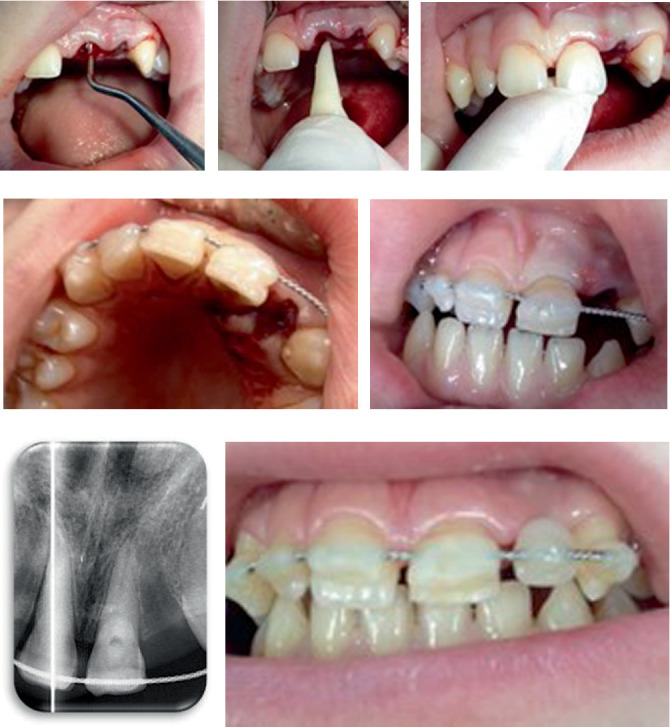
Replanting Tooth 21 after avulsion: (a) preparation of the alveolus; (b) insertion of the tooth into the alveolus; (c) appearance immediately after replanting; (d) immobilization of neighboring Teeth 13, 12, 11, and 13 (palatal view); (e) adaptation of the occlusion; (f) Rx after replanting and immobilization; and (g) clinical appearance 4 weeks after the intervention.

The replantation was performed on an outpatient basis. After administering local anesthesia, the alveolar socket was reexamined (Figure 3), and the blood clot was gently removed through careful curettage and saline irrigation. With light digital pressure, the tooth was reinserted into the alveolus (Figures 3 and 3), and its position was stabilized using a custom-fabricated silicone template. The tooth was then splinted to the adjacent teeth using flexible wire and composite resin (Figures 3 and 3). Radiographic evaluation (Figure 3) confirmed the accurate positioning of the tooth in the alveolus. Following the splinting procedure, both static and dynamic occlusal contacts of Tooth 2.1 were verified to avoid any interference. For esthetic purposes, a composite tooth was attached to the flexible wire to mask the absence of the lateral incisor (Figure 3).

### 2.2. Surgical Stage and Postoperative Management

**Figure 4:**
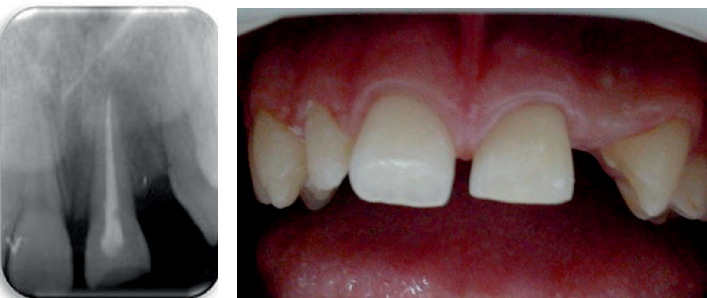
The postoperative control: (a) canal filling and (b) clinical appearance 6 weeks after replanting.

Postoperatively, ampicillin was prescribed at 1 g every 12 h for 5 days, along with ibuprofen 400 mg twice daily for its anti-inflammatory effect (for a maximum of 5 days). A soft diet was recommended for 14 days, and the patient was instructed to practice meticulous oral hygiene. The postoperative evaluation on the second day demonstrated normal healing. It was recommended that the splint be maintained for 4 weeks. At the 1-month follow-up, the patient reported a favorable evolution with no pain or signs of inflammation. After splint removal at 4 weeks, neither abnormal tooth mobility nor a positive response to percussion was detected. The intracanal dressing, consisting of calcium hydroxide paste, was refreshed, and root canal filling was performed (Figure 4a) 6 weeks after replantation by lateral condensation using a 0.4 ISO 40 master cone and Sealapex (Kerr Dental). Tooth 2.1 is within normal limits on clinical examination 6 weeks after replantation (Figure 4b).

### 2.3. Follow-Up and Long-Term Monitoring

The patient and his parents were informed of the necessity for an orthodontic evaluation to maintain the space corresponding to Tooth 2.2, although this plan has not yet been implemented. Patient monitoring was scheduled at 6-month intervals. At the 1-year follow-up, Tooth 2.1 remained asymptomatic and exhibited a stable, functional position; periodontal probing was intentionally avoided to prevent further trauma.

**Figure 5:**
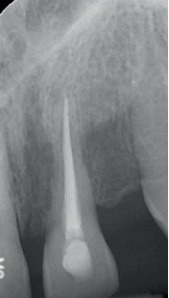
Rx after 1 year: areas of inflammatory external root resorption are observed along the mesial root surface and in the distal middle third.

Unfortunately, no measures were taken to restore the edentulous space resulting from the avulsion of the lateral incisor, and vitality tests of adjacent teeth yielded normal responses. However, intraoral radiographs obtained at the 1-year follow-up revealed signs of inflammatory resorption along the root of the replanted tooth (Figure 5). Given the potential for future complications in this borderline case, the follow-up period was extended. At the 18-month evaluation, inflammatory resorption continued to be observed along the root of Tooth 2.1. Regrettably, the patient did not return for this appointment, and his parents later informed us that the entire family had emigrated.

## 3. Discussions

Tooth avulsion is a serious trauma that predominantly affects the maxillary incisors. Replantation of avulsed teeth involves recovery from the accident site and placement in a physiological storage environment for PDL cells until presentation to the dentist. The International Association of Dental Traumatology (IADT) differentiates the therapeutic approach depending on the transport environment in the period of time from avulsion to replantation and the duration of this period, correlated with the degree of development of the avulsed tooth root. For a period longer than 60 min in an extraoral environment, replantation is no longer recommended [4]. Replantation of avulsed teeth involves several stages of treatment and monitoring.

The root of the teeth is physiologically protected against resorption with a layer of unmineralized cement. The number of surviving PDL cells is determined by the regenerative capacity of this tissue [5]. Depending on the severity of the trauma and the posttraumatic situation (time and means of transport), the nonmineralized cement layer can no longer act as a barrier against the clastic cells in the adjacent bone [6]. Root resorption is performed by osteoclasts, which are multinucleated cells derived from the phagocytic system, which colonizes denuded mineralized tissues and initiates resorptive responses [7]. Therefore, after replantation due to the occurrence of external root resorption, it is to be expected.

External surface resorption is a self-limiting phenomenon typically triggered by a localized injury to the affected area of the cementum and/or PDL. It can be viewed as a component of the healing response after a minor injury [8]. When distinct regions experience PDL necrosis, ankylosis develops in those locations. At first, ankylosis does not involve resorption; instead, the tooth root and the alveolar bone come into direct contact and seem to be fused together. In favorable cases on demarcated, small areas, PDL and cement are replaced with bone tissue for a limited time. Even in the presence of ankylosis, the prognosis is acceptable [6]. Ultimately, and with varying durations based on the cause of the ankylosis, this will result in external replacement resorption [8]. External replacement root resorbtion (ERR) or external inflammatory resorption (EIR) are expected adverse outcomes [5].

EIR triggered by harmful factors like bacterial infections, necrotic cellular debris, and mechanical stresses can result in quick tooth loss [6]. Furthermore, investigations on animals have demonstrated that damage to more than 20% of the PDL on the root surface and flaws larger than 4 mm^2^ result in a poor prognosis for replantation [7].

External replacement resorption (ERR) is the process by which cementum and dentin are resorbed under the action of osteoblasts and replaced with alveolar bone. The intensity, duration, and advancement of these resorptive processes (ERR) result in the substitution of root tissues with alveolar bone, ultimately leading to the loss of the dental crown [7, 8].

A meta-analysis of published data regarding external root resorption found that the incidence of ERR was 51%, EIR was 23.2%, and surface root resorption was 13.3% [9].

The IADT guidelines suggest that an avulsed tooth should be replanted within 1 h following the injury, as the health of the PDL becomes uncertain after 60 min, regardless of how it is stored [4, 10].

Contrary to the IADT guidelines addressing the extraoral interval during which dental replantation is recommended, the extraoral period in the case at hand is roughly 30 h.

In terms of storage conditions, we valued the tooth's hydration up until the point of recovery. Unfortunately, the weakest storage environment for avulsed teeth is thought to be water. In the situation described, the tooth was stored in milk from the moment it was recovered until it was replanted, with the intention of preserving the vitality of the remaining PDL cells. Additionally, during this extended time at ground level, the dento-PDLs became polluted. Given the facts of the instance at hand, the concessions made in relation to the IADT recommendations about replantation can be used to explain why resorption occurred a year following replantation.

Milk is regarded as the most suitable storage medium today because of its availability, along with its favorable chemical and biological characteristics that help keep PDL cells viable [4, 10]. Various storage media have been tested over time, and after milk, saliva and saline are storage media considered by IADT [4, 10–12].

Several clinical studies have examined the connection between the storage conditions of an avulsed tooth, the duration it remains outside the mouth before replantation, and the development of resorptive processes and ankylosis. In this context, the research on the likelihood of ankylosis following the storage of permanent teeth with fully developed apexes in saliva, carried out by Albertsson et al., found that for every extra minute teeth were kept in saliva, the risk of developing ankylosis increased by 1% [13].

A retrospective study comprising 196 replanted teeth that were maintained for 30 min in a nonphysiological setting demonstrated that this time frame is crucial for the periodontal repair of a replanted tooth [14]. After an average of 4 years, an analysis of replanted teeth showed that EIR occurred in 23% of instances and ERR in 55.6% of cases [8]. According to specialized research, juvenile patients (6–16 years old) experience a faster rate of resorption progression following delayed tooth replantation than do adult patients (17–39 years old) [7]. For mature teeth with closed apices, the rate of tooth resorption following delayed replantation appears to be slower [7].

Careful management of the tooth and treatment of the root surface prior to replantation, along with the commencement of endodontic therapy, is crucial in avoiding resorptive processes. The IADT guidelines suggest that endodontic treatment should take place after the tooth has been replanted and stabilized [4]. In this case, the endodontic procedure was conducted outside the mouth, as the clinical circumstances did not permit sufficient isolation following immobilization.

Endodontic therapy should begin within 2 weeks following replantation, and an intracanal medicament should be utilized as an antimicrobial agent to avert the occurrence of EIR (4, 8, and 15). Pastes that contain antibiotics and corticosteroids, such as Ledermix or Odontopaste (ADM, Brisbane, Australia), which have antiresorptive effects, are recommended, followed by the application of calcium hydroxide [4, 15]. Since the endodontic procedure was conducted outside the mouth prior to the replantation, we utilized calcium hydroxide inside the canal. We focused on the movement of hydroxyl ions through the dentinal tubules toward the root surface to promote PDL healing [15]. One option could have been to permanently seal the root canal prior to replantation, as noted in comparable cases [16].

In the provided example, resorption took place a year following replantation, suggesting an elevated occurrence rate. Factors surrounding the replantation, such as delay, contamination, poor storage conditions, wide apex, and the age of the patient, contribute to this situation. Besides the treatment, the dentist must consider the possible ramifications, including survival rates, the effects of replantation on future procedures, and the costs associated with the choice to replant a tooth [17].

When dealing with oral trauma, the information is taken in while the patient is experiencing a significant emotional strain. Consequently, the patient's decision remained unchanged despite the cautious prognosis being shared in this case. Unfortunately, the resolution was not maintained throughout the observation period due to valid reasons.

A risk that arises during the prepubertal stage in which the patient is currently situated is the restriction of growth in the corresponding alveolar bone process [8, 13, 18]. M. Tsukiboshi and T. Tsukiboshi [19] observed considerable morphological changes in the alveolar bone after delayed replantation of avulsed teeth with subsequent ankylosis, in patients under 18 years of age. Bone resorption is significant vestibularly compared to palatal resorption. Other research reported an infraposition of ankylosed maxillary incisors at a rate of 0.42 mm per year in 16-year-old boys [6]. Annual monitoring for a period of 5 years, recommended by the IADT, facilitates a therapeutic course appropriate to the development during the growth period and beyond.

Dentists handle cases of dental trauma by applying the knowledge acquired during their training and adhering to the best practice guidelines set by the IADT or related national standards [20]. Regarding professional information, the decision to postpone replantation warrants thorough investigation, and treatment information should be consistently revised. In contrast to the 4 weeks that were advised at the time the case was treated, the current suggestion is to continue immobilization for 2 weeks [4, 12, 13].

Individuals who have experienced trauma, regardless of their age, arrive at the dental clinic seeking reimplantation of teeth that have been knocked out. Distressed and in shock from the incident, they place their full trust in reimplantation, which is viewed as a sign of hope for success. Even when dentists have reservations about the likely outcome, they still carry out delayed reimplantation. In the heat of the moment, healthcare providers are seen as rescuers, and the grim predictions are often pushed aside. Eventually, as resorptive changes become apparent months or even years postreplantation, physicians may encounter complaints. Initially, feelings of enthusiasm and thankfulness can shift into criticism and disappointment when the results are not favorable.

However, not replanting an avulsed tooth is an irreversible decision [6], and the dilemma generated by the questionable prognosis is ignored. The avulsed tooth must be replanted to prevent possible subsequent complaints in the event of not replanting an avulsed tooth [9]. Individuals seeking information online, including patients and their parents, educate themselves about first aid and the process of replanting an avulsed tooth. There is a lack of information regarding the uncertain developments following replantation [9]. The success associated with the reattachment of an avulsed tooth is seen as an unquestionable fact.

Delayed dental replantation has an uncertain long-term prognosis and may be considered a temporary treatment, especially in young patients. Preservation of the tooth and bone tissue favors optimal development of the alveolar process until other restorative options, such as dental implants, can be considered for later restoration.

## 4. Conclusions

Despite the inherent risks, the replantation of avulsed teeth remains the treatment of choice, particularly in prepubertal patients where preserving alveolar bone growth is critical. Even though accelerated resorption and subsequent complications can occur, replantation may still significantly alleviate the psychological, esthetic, and social stresses associated with dental trauma.

## Data Availability

Data sharing is not applicable to this article.
